# Case Report: Wolman disease caused by LIPA variants in two Chinese infants and a focused literature synthesis

**DOI:** 10.3389/fped.2026.1933873

**Published:** 2026-08-20

**Authors:** Leping Zhang, Qiong Yu, Xinjing Ge, Xinbao Xie

**Affiliations:** 1 Department of Pediatrics, Sijing Hospital of Songjiang District, Shanghai, China; 2 Department of Pediatrics, Inner Mongolia Maternal and Child Health Care Hospital, Hohhot, China; 3 Pediatric Liver Center, Children’s Hospital of Fudan University, National Children’s Medical Center, Shanghai, China

**Keywords:** adrenal calcification, case report, infantile hepatosplenomegaly, LIPA, lysosomal acid lipase deficiency, Wolman disease

## Abstract

**Background:**

Wolman disease is the severe infantile form of lysosomal acid lipase deficiency, caused by biallelic pathogenic variants in the Lysosomal Acid Lipase (LIPA) gene. It is rapidly progressive and often fatal within the first year of life without disease-specific therapy. Because early manifestations are non-specific, timely diagnosis remains challenging.

**Case presentation:**

We report the cases of two Chinese male infants with genetically confirmed Wolman disease. Case 1 involved an 8-week-old infant—born to consanguineous parents—who presented with persistent diarrhea, recurrent low-grade fever, abdominal distension, hepatosplenomegaly, ascites, bilateral adrenal involvement, anemia, thrombocytopenia, hypoproteinemia, dyslipidemia, coagulopathy, and electrolyte disturbances. Liver biopsy showed diffuse hepatocyte vacuolization. Genetic testing identified a novel homozygous LIPA variant—c.285G > T/p.Trp95Cys—inherited from heterozygous carrier parents. Lysosomal acid lipase activity was markedly reduced to 0.62% of the normal control level. Case 2 involved an 11-week-old infant who presented with failure to thrive, poor feeding, abdominal distension, cough, progressive hepatosplenomegaly, ascites, bilateral adrenal calcifications, hepatic dysfunction, systemic inflammation, coagulopathy, anemia, and thrombocytopenia. He carried compound heterozygous LIPA non-sense variants, c.796G > T/p.Gly266* and c. 193C > T/p.Arg65*, inherited from his mother and father, respectively. Despite supportive care, both infants developed progressive multiorgan failure and died.

**Conclusion:**

These cases expand the clinical and molecular spectrum of Wolman disease in Chinese infants and highlight important diagnostic challenges, including non-specific early manifestations and inflammatory presentations mimicking hematologic disorders. Recognition of the characteristic combination of hepatosplenomegaly, failure to thrive, dyslipidemia, adrenal calcification, reduced lysosomal acid lipase activity, and LIPA variants may facilitate early diagnosis. Timely identification is essential because early enzyme replacement therapy may alter the otherwise fatal natural course of this rapidly progressive disease.

## 1 Introduction

Lysosomal acid lipase deficiency is an autosomal recessive lysosomal storage disorder caused by pathogenic variants in the Lysosomal Acid Lipase (LIPA) gene, which encodes lysosomal acid lipase. This enzyme hydrolyses cholesteryl esters and triglycerides within lysosomes. Severe loss of lysosomal acid lipase activity causes progressive intracellular accumulation of neutral lipids, particularly in the liver, spleen, adrenal glands, bone marrow, intestine, and reticuloendothelial system (1–4).

The clinical spectrum ranges from the severe infantile phenotype, Wolman disease, to the later-onset and usually milder phenotype, cholesteryl ester storage disease. Wolman disease typically presents during the first weeks or months of life with vomiting, diarrhea, failure to thrive, hepatosplenomegaly, liver dysfunction, dyslipidemia, malabsorption, anemia, thrombocytopenia, and adrenal calcifications (3–5). Untreated infants usually die within the first year of life from liver failure, infection, bleeding, malnutrition, or multiorgan failure (4, 6).

Early diagnosis remains challenging because Wolman disease is ultra-rare and its initial manifestations overlap with neonatal cholestasis, infection, hemophagocytic lymphohistiocytosis, leukemia-like presentations, and other inherited metabolic diseases (7–9). Here, we report the cases of two Chinese infants with Wolman disease, including one carrying a novel homozygous LIPA variant, and provide a focused literature synthesis to support earlier recognition of this treatable but rapidly fatal pediatric metabolic disorder.

## 2 Case description

Case 1 involved an 8-week-old Chinese boy admitted to the hospital on 12 January 2016, with watery diarrhea for 20 days, occurring at least three times daily. He was the second child of consanguineous parents, who were second cousins. His mother had two previous pregnancies terminated during early gestation because of fetal growth arrest. The patient was born at term by Cesarean section after an otherwise uneventful pregnancy, with a birth weight of 4,400 g. Neonatal jaundice developed on day 4 after birth, followed by recurrent low-grade fever.

At admission, his weight was 5.0 kg and length was 50 cm. His abdominal circumference was 46 cm, reflecting marked abdominal distension associated with hepatosplenomegaly and ascites. Physical examination revealed marked abdominal distension, bilateral scrotal swelling, hepatomegaly, and splenomegaly, consistent with progressive visceral involvement characteristic of Wolman disease. Laboratory investigations demonstrated anemia, thrombocytopenia, hypoproteinemia, hypoalbuminemia, mild hepatic injury, dyslipidemia, coagulopathy, and multiple electrolyte abnormalities. Hemoglobin ranged from 64.0 to 112.0 g/L, platelet count from 45 to 131 × 10^9^/L, albumin from 15.4 to 34.0 g/L, and triglycerides from 0.43 to 3.03 mmol/L. Alpha-fetoprotein was markedly elevated at 1,156 ng/mL.

**Figure 1:**
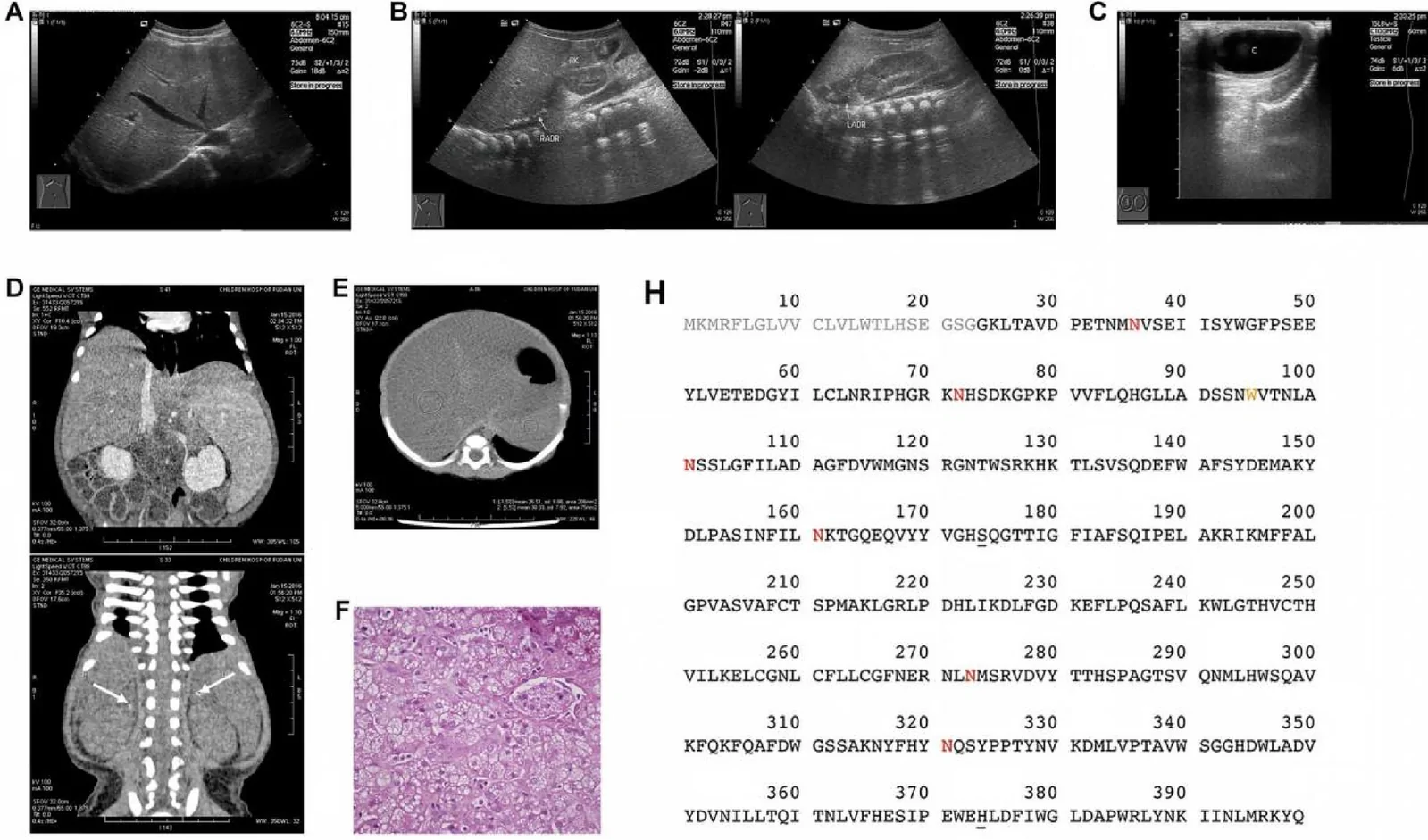
Imaging, histopathological, and genetic findings in Case 1. **(A)** Abdominal ultrasonography showing hepatosplenomegaly. **(B)** Abdominal ultrasonography showing bilateral adrenal abnormalities. **(C)** Testicular ultrasonography showing bilateral hydroceles. **(D)** Upper abdominal computed tomography showing bilateral adrenal calcifications (arrows). **(E)** Liver computed tomography showing severe hepatic steatosis with markedly reduced hepatic attenuation. **(F)** Liver biopsy showing diffuse hepatocyte vacuolization and portal inflammatory cell infiltration. **(H)** Schematic representation of lysosomal acid lipase protein structure and the position of the p.Trp95Cys variant.

Abdominal ultrasonography revealed progressive hepatosplenomegaly, hepatic parenchymal abnormalities, mild ascites, heterogeneous bilateral adrenal enlargement, and bilateral testicular hydroceles (Figures 1A–C). Abdominal computed tomography confirmed bilateral adrenal calcifications and severe hepatic steatosis, with markedly reduced liver attenuation (Figure 1D). Liver biopsy demonstrated diffuse hepatocyte vacuolization with inflammatory infiltration in the portal tracts (Figure 1E). Genetic testing identified a homozygous LIPA variant, c.285G > T, resulting in p.Trp95Cys. Segregation analysis confirmed that both parents were heterozygous carriers. This variant was not found in the reviewed literature and was interpreted as a novel candidate pathogenic variant in the clinical context of severe infantile lysosomal acid lipase deficiency. Lysosomal acid lipase activity was markedly reduced to 0.62% of the normal control level. The patient received comprehensive supportive management, including anti-infective therapy, gastrointestinal decompression, plasma, platelet and red blood cell transfusions, albumin supplementation, intravenous immunoglobulin administration, and electrolyte correction to maintain metabolic stability. Despite these interventions, the disease progressed rapidly, resulting in multiorgan failure and death (Table 1).

**Table 1:** Clinical timeline and diagnostic summary of the two infants with Wolman disease.

Clinical item	Case 1	Case 2
Age and sex	8-week-old male infant	11-week-old male infant
Family background	Consanguineous parents; two previous early pregnancy losses due to fetal growth arrest	Non-consanguineous parents; older brother reportedly healthy
Birth history	Term Cesarean section; birth weight 4,400 g	Term Cesarean section because of prolonged labor; birth weight 3,550 g
Initial symptoms	Watery diarrhea for 20 days; recurrent low-grade fever; neonatal jaundice after birth	Failure to thrive, poor appetite, abdominal distension, sleep disturbance, and persistent cough
Key examination findings	Marked abdominal distension, bilateral scrotal swelling, hepatomegaly, and splenomegaly	Severe abdominal distension, bilateral scrotal swelling, hepatomegaly, and splenomegaly
Laboratory abnormalities	Anemia, thrombocytopenia, hypoproteinemia, hypoalbuminemia, dyslipidemia, coagulopathy, and electrolyte disturbances	Anemia, thrombocytopenia, hepatic dysfunction, cholestasis, hypoproteinemia, coagulopathy, and systemic inflammation
Imaging findings	Progressive hepatosplenomegaly, ascites, bilateral adrenal abnormalities/calcifications, testicular hydroceles, and hepatic steatosis	Pulmonary congestion and pleural effusion, hepatosplenomegaly, ascites, bilateral adrenal calcifications, and abdominal distension
Pathology/marrow	Liver biopsy: diffuse hepatocyte vacuolization with portal inflammatory infiltration	Bone marrow: hypercellularity, dysplastic megakaryocytes, and occasional hemophagocytic cells
Genetic findings	Homozygous LIPA c.285G > T/p.Trp95Cys; both parents heterozygous carriers	Compound heterozygous LIPA c. 796G > T/p.Gly266* and c.193C > T/p.Arg65*; maternally and paternally inherited, respectively
Lysosomal acid lipase (LAL) activity	0.62% of the normal control level	Not available
Treatment	Anti-infective therapy, gastrointestinal decompression, plasma transfusion, platelet transfusion, red blood cell transfusion, albumin supplementation, intravenous immunoglobulin administration, and electrolyte correction	Dietary modification, antibiotics, albumin supplementation, and supportive care
Outcome	Death from progressive multiorgan failure	Death from progressive multiorgan failure after withdrawal of care by the family

Case 2 involved an 11-week-old Chinese boy admitted to the hospital in January 2020 because of failure to thrive, poor appetite, abdominal distension, sleep disturbance, and persistent cough. He was the second child of healthy, non-consanguineous Chinese parents. He was born at term by Cesarean section because of prolonged labor, with a birth weight of 3,550 g. His older brother was reportedly healthy.

**Figure 2:**
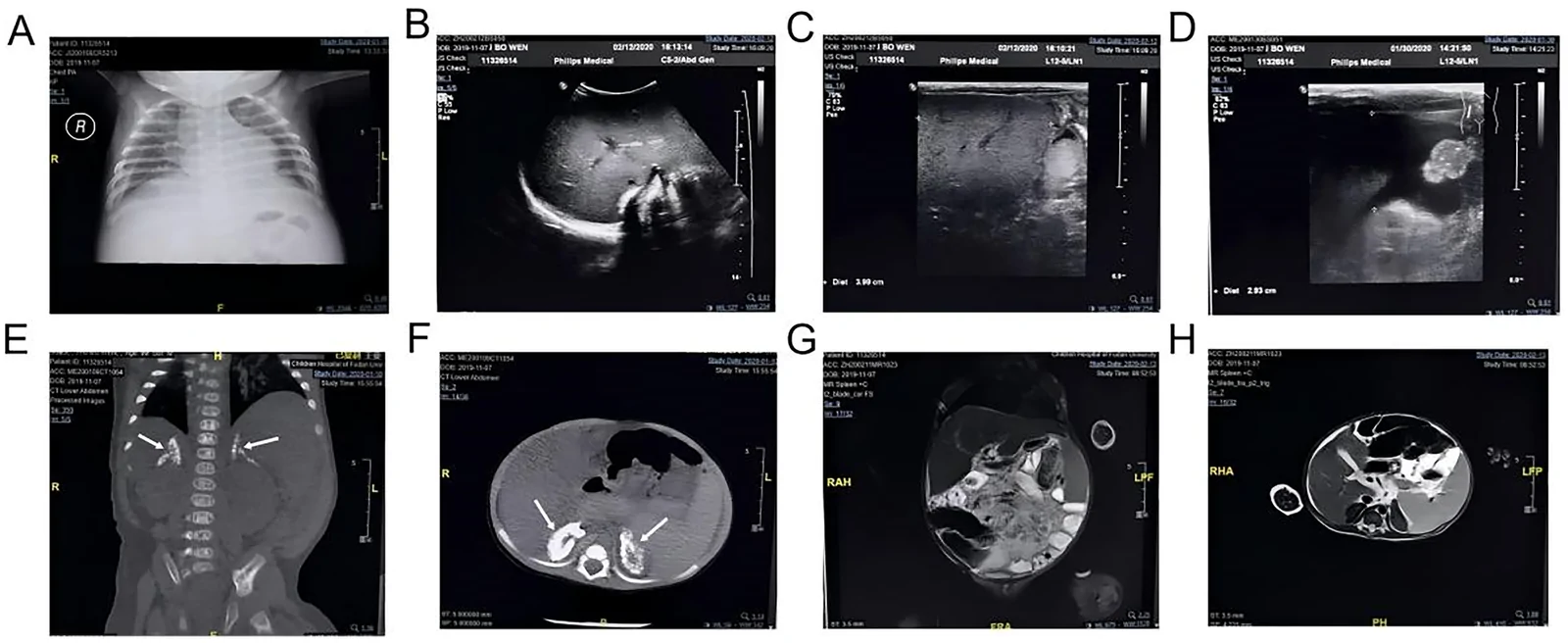
Imaging findings in Case 2. **(A)** Chest radiograph showing pulmonary congestion and pleural effusion. **(B)** Abdominal ultrasonography showing hepatomegaly. **(C)** Abdominal ultrasonography showing splenomegaly. **(D)** Abdominal ultrasonography showing ascites. **(E)** Abdominal computed tomography showing hepatosplenomegaly, abdominal distension, and bilateral adrenal calcifications (arrows). **(F)** Computed tomography demonstrating bilateral adrenal calcifications (arrows). **(G)** Abdominal magnetic resonance imaging showing marked abdominal distension and organomegaly. **(H)** Magnetic resonance imaging focused on the liver and spleen.

At presentation, his weight was 5.0 kg and length was 53 cm. His abdominal circumference was 45 cm, consistent with severe abdominal distension caused by hepatosplenomegaly and ascites. Physical examination revealed severe abdominal distension, bilateral scrotal swelling, hepatomegaly 3.5 cm below the right costal margin, and splenomegaly 2 cm below the left costal margin. Chest radiography showed pulmonary congestion and pleural effusion (Figure 2A). Abdominal ultrasonography demonstrated progressive hepatosplenomegaly and ascites (Figures 2C,D). Abdominal computed tomography revealed bilateral adrenal calcifications and hepatosplenomegaly (Figures 2E,F). Magnetic resonance imaging confirmed marked hepatosplenomegaly and abdominal distension (Figures 2G,H).

Laboratory investigations revealed anemia, thrombocytopenia, hepatic dysfunction, cholestasis, hypoproteinemia, coagulopathy, and systemic inflammation. Hemoglobin ranged from 79 to 107 g/L, platelet count from 64 to 218 × 10^9^/L, alanine aminotransferase from 47 to 76 U/L, aspartate aminotransferase from 144 to 235 U/L, total bilirubin from 46 to 162 μmol/L, direct bilirubin from 33 to 128 μmol/L, and albumin from 28 to 39 g/L. The international normalized ratio increased to 1.75–2.05. C-reactive protein remained elevated at 27–86 mg/L. Bone marrow aspiration revealed hypercellular marrow, decreased myeloid-to-erythroid ratio, dysplastic megakaryocytes, and occasional hemophagocytic cells.

Genetic analysis identified compound heterozygous LIPA variants: c.796G > T/p.Gly266* inherited from the mother and c. 193C > T/p.Arg65* inherited from the father. Both variants introduce premature termination codons and have been previously reported as pathogenic. The infant received dietary modification, broad-spectrum antibiotics, albumin supplementation, and other supportive measures. After transient marginal improvement, the family requested discharge. One week later, he developed lethargy, worsening hypoproteinemia, gastrointestinal bleeding, epistaxis, refractory cytopenia, and progressive multiorgan failure. Care was withdrawn by the family, and the infant died shortly thereafter (Table 1).

## 3 Diagnostic assessment, therapeutic intervention, follow-up, and outcomes

In both infants, Wolman disease was suspected based on the combination of early infancy onset, failure to thrive or persistent diarrhea, progressive hepatosplenomegaly, ascites, cytopenia, hepatic dysfunction, dyslipidemia, hypoproteinemia, coagulopathy, and adrenal involvement. Bilateral adrenal calcification was particularly important in narrowing the differential diagnosis. The diagnosis was confirmed by LIPA genetic testing and, in Case 1, by severely deficient lysosomal acid lipase activity.

Neither infant received disease-specific enzyme replacement therapy. Detailed supportive interventions for each patient are described in the corresponding case presentations. Despite supportive management, both infants experienced rapid disease progression and died from multiorgan failure (Table 1), consistent with the severe natural history of untreated Wolman disease.

## 4 Focused literature synthesis

A focused literature search was performed in PubMed and CNKI using the terms “lysosomal acid lipase,” “Wolman disease,” “Wolman's disease,” “lysosomal acid lipase deficiency,” “lysosomal storage disease,” and “lipase A.” Reports with pediatric Wolman disease and available LIPA genotypic information were reviewed. This descriptive synthesis was used to contextualize the present cases clinically and genetically rather than to perform a formal systematic review.

Across 76 previously reported pediatric patients, the median age at onset or diagnosis was approximately 60 days. The most frequent clinical features were hepatomegaly, splenomegaly, failure to thrive, adrenal calcification, abdominal distension, diarrhea, and vomiting. Common laboratory abnormalities included anemia, elevated transaminases, hypertriglyceridemia, coagulopathy, hypoalbuminemia, and hyperbilirubinemia. Forty-nine distinct LIPA variants were identified, including missense, non-sense, splice-site, deletion, insertion, and large deletion variants. The splice-site variant c.966 + 2T > G was among the most frequently reported variants, particularly in Spanish patients (10). Among patients with known outcomes, untreated or supportively treated infants had very poor survival, whereas long-term survival has been described after early enzyme replacement therapy, hematopoietic stem cell transplantation, or combined approaches (6, 11–18).

## 5 Discussion

This report describes two genetically confirmed Chinese infants with Wolman disease and provides comprehensive clinical, biochemical, and molecular characterization of this ultra-rare disorder. Although the number of patients is limited due to the rarity of Wolman disease, detailed characterization of individual cases remains valuable for improving disease recognition and expanding the understanding of its clinical and molecular spectrum. The present cases highlight three clinically important aspects of Wolman disease: early recognition of characteristic diagnostic clues, genotype–phenotype correlation, and the importance of timely disease-specific intervention.

First, the clinical pattern was highly consistent with severe infantile lysosomal acid lipase deficiency. Both infants presented within the first 3 months of life and showed rapidly progressive hepatosplenomegaly, abdominal distension, ascites, hepatic dysfunction, hypoproteinemia, cytopenia, and coagulopathy. Bilateral adrenal calcification was a decisive diagnostic clue. In an infant with failure to thrive, hepatosplenomegaly, diarrhea or feeding difficulty, dyslipidemia, and adrenal calcification, Wolman disease should be considered early and tested promptly by lysosomal acid lipase activity assay and LIPA sequencing.

Second, Case 2 highlights an under-recognized inflammatory phenotype of Wolman disease. The presence of persistent systemic inflammation, cytopenia, and occasional hemophagocytic cells in the bone marrow further illustrates the clinical heterogeneity of this disorder. Previous reports have shown that Wolman disease can present with secondary hemophagocytic lymphohistiocytosis-like features, including fever, cytopenia, hepatosplenomegaly, hypertriglyceridemia, and inflammatory activation (7, 9, 19). These overlapping manifestations may delay metabolic diagnosis. Therefore, in infants with suspected hemophagocytic lymphohistiocytosis but accompanied by adrenal calcification, severe hepatosplenomegaly, dyslipidemia, malabsorption, or unexplained liver failure, lysosomal acid lipase deficiency should remain in the differential diagnosis.

Third, the novel c.285G > T/p.Trp95Cys variant expands the known LIPA mutational spectrum. The substituted residue lies within the mature lysosomal acid lipase protein, and replacement of tryptophan by cysteine may disturb local folding, protein stability, or catalytic-domain conformation. The severe phenotype, homozygous state, parental carrier segregation, and enzyme activity of only 0.62% of the normal control level support pathogenicity. Nevertheless, functional validation in expression systems would strengthen molecular interpretation. By contrast, the two variants in Case 2, p.Gly266* and p.Arg65*, are non-sense variants expected to cause truncated, non-functional protein or non-sense-mediated mRNA decay, consistent with severe infantile disease.

Genotype–phenotype relationships in lysosomal acid lipase deficiency are not fully linear. Residual enzyme activity is a major determinant, but phenotype may also depend on transcript processing, protein folding, allelic combination, variant position, non-sense-mediated decay, and possible modifier factors. Some variants or regional splice effects can be associated with different residual activity levels and clinical phenotypes (10, 20–25). Clinical interpretation should therefore integrate genotype, enzyme activity, age at onset, organ involvement, and disease trajectory rather than relying on variant class alone.

The prognosis of untreated Wolman disease remains poor. Supportive care, nutritional modification, and infection control may provide temporary stabilization but do not correct the underlying lysosomal lipid storage (26). Enzyme replacement therapy with sebelipase alfa has changed the therapeutic landscape and can improve survival, growth, hepatic function, gastrointestinal symptoms, and systemic status when initiated early (15–18). Hematopoietic stem cell transplantation has also produced long-term survival in selected patients, although transplant-related morbidity and mortality remain important concerns (11–14). Current evidence supports rapid diagnosis followed by early disease-specific therapy, with individualized consideration of enzyme replacement therapy, nutritional support, and transplantation strategies in specialized centers. In addition, gene therapy may represent a potential curative strategy (27).

The main strength of this report lies in the comprehensive integration of clinical, imaging, histopathological, enzymatic, and genetic findings from two infants with genetically confirmed Wolman disease. The identification of a novel homozygous LIPA variant, supported by parental segregation analysis and markedly reduced lysosomal acid lipase activity, provides additional information for the molecular characterization of this disorder. Furthermore, the focused literature synthesis offers broader clinical and genetic context for interpreting the present findings. This study has several limitations, including its retrospective nature, the small number of cases inherent to the rarity of Wolman disease, incomplete enzyme activity data in Case 2, and the absence of *in vitro* functional validation for p.Trp95Cys.

## 6 Patient perspective

Because both patients were infants and died during early disease progression, direct patient perspectives could not be obtained. The legal guardians were informed of the diagnostic findings, disease severity, expected prognosis, and available management considerations. Written informed consent for publication of the clinical details and images was obtained from the legal guardians.

## 7 Conclusion

We reported the cases of two Chinese infants with genetically confirmed Wolman disease, including one carrying a novel homozygous LIPA variant, c.285G > T/p.Trp95Cys, associated with profoundly reduced lysosomal acid lipase activity. These cases reinforce the importance of recognizing the diagnostic constellation of early-onset gastrointestinal symptoms, growth failure, hepatosplenomegaly, dyslipidemia, liver dysfunction, cytopenia, and adrenal calcifications. Early biochemical and genetic confirmation is essential because Wolman disease is rapidly fatal without disease-specific therapy, whereas timely enzyme replacement therapy may alter the natural course.

## Data Availability

The original contributions presented in the study are included in the article/Supplementary Material; further inquiries can be directed to the corresponding author.
