# Supplementary material for: Case Report: Wolman disease caused by LIPA variants in two Chinese infants and a focused literature synthesis
Source: Front Pediatr. 2026 Aug 20;14:1933873. doi: 10.3389/fped.2026.1933873 (PMC13538864; doi:10.3389/fped.2026.1933873)
Supplement: Supplementary file 1 [file Table1.docx]

**Supplementary Table	S1.** Reported pediatric Wolman	disease	cases with	available LIPA genotypic information.

| **Patient** | **Country/Region** | **Genotype** | **Zygotic type** | **Age at onset/diagnosis** | **Gender** | **Consanguinity** | **Clinical manifestation** | **Lab findings** | **Biopsy** | **RLALa** | **Therapy** | **Prognosis** | **Death** | **Ref.** |
| --- | --- | --- | --- | --- | --- | --- | --- | --- | --- | --- | --- | --- | --- | --- |
| 1 | America | c.634-635insT(P212Lfs*14)/c.638T＞C(p.L179P) | CH | ND | ND | NO | FTT, HM, AC | ND | ND | + | ND | death | 6.5 months | [31] |
| 2 | America | c.634-635insT(P212Lfs*14)/c.638T＞C(p.L179P) | CH | ND | ND | NO | FTT, HM, AC | ND | ND | ND | ND | death | 3 months | [31] |
| 3 | Japan | c.129C＞G(p.Y43*) | H | 30 days | F | YES | D, V, FTT, HSM, AD, AC | A, ET | ND | + | ND | death | 114 days | [32] |
| 4 | Mali | c.892C>T/(p.Q298*) | H | 2 months | M | YES | FTT, HSM, AC | A | ND | + | ND | Alive at 12 months |  | [16] |
| 5 | Mali | E8SJM-3(delta 254-277) | H | ND | F | YES | ND | ND | ND | ND | ND | death | 5 months | [16] |
| 6 | Germany | E8SJM+1(delta 254-277) | H | ND | ND | YES | ND | ND | ND | + | ND | death | 3 months | [15] |
| 7 | Germany | E8SJM+1(delta 254-277) | H | ND | ND | YES | ND | ND | ND | + | ND | death | 6 months | [1][15] |
| 8 | Italy | 1012A＞T(p.Y303*) | H | 1 month | M | ND | D, FTT, HM, AC | ND | ND | + | ND | death | 8 months | [33] |
| 9 | Germany | c.119_127del8 (p.G24*)/c.395_397del2(p.W116*) | CH | 1 month | F | NO | D, V, FTT, HSM, AC, AD | A, ET, CG | Lb，BMb | + | ST | death | 11 Weeks | [34] |
| 10 | Turkish | c.1024G＞T(p.G342W) | H | 40 days | F | YES | D, V, FTT, HSM, AD, AC | A, ET, HTG, CG | BMb | + | ST | death | 9 Weeks | [34] |
| 11 | Caucasian | Exon4 skipped/Undetermined | CH | 2 months | M | NO | ND | ND | Lb，BMb | + | ND | death | 4 months | [19] |
| 12 | America | c.347G＞A(p.W116*)/c.684delT (p.Phe22Leufs*13) | CH | 3 months | M | NO | ND | ND | Lb，BMb | + | ND | death | 4 months | [19] |
| 13 | Africa | c.594-595insT(p.A199Cfs*13)/c.193C＞T(p.R65*) | CH | 2 months | F | ND | D, V, FTT, HM | ND | ND | + | ND | death | 3.5 months | [19] |
| 14 | America | 347G＞A/(p.W116* )/724delT(p.W242Gfs*12) | CH | 1 month | F | ND | D, V, FTT, AC | A, HTG, HB | Lb | + | BMT | Alive at 36 months |  | [24] |
| 15 | Israel | c.398delC(p. S133*) | H | ND | ND | YES | D, HSM, AC | A | ND | + | ND | ND | 12 Weeks | [35] |
| 16 | Israel | c.398delC(p. S133*) | H | ND | F | NO | HSM, AC | ND | ND | + | ND | ND | 3 months | [35] |
| 17 | Uzbekistan | c.67G＞A(p.G23R)/c.260G＞T(p.G87V) | CH | ND | ND | YES | HSM, AC | ND | ND | + | ND | ND | 12 Weeks | [35] |
| 18 | Uzbekistan | c.67G＞A(p.G23R) | H | ND | F | YES | D, V, FTT, HSM, AD, AC | ND | ND | + | UMCBT | Alive at 4 months |  | [23] |
| 19 | America | c.401G＞A(p.V134G) /c.836T＞G(p.V279G ) | CH | 50 days | F | ND | D, FTT, HSM, AD, AC | ND | ND | + | HSCT |  | 158 days | [25] |
| 20 | America | c.482delA(p.N161Ifs*19)/exon 4 del | CH | 6 Weeks | M | NO | V, FTT, HSM, AD, AC | ND | ND | + | ST | death | 10 Weeks | [36] |
| 21 | China | c.318insT(p.Phe106fsX4) | H | 1Week | M | NO | D, V, FTT, HSM, AD, AC | CG, HB | Lb | + | ST | death | 4 Weeks | [37] |
| 22 | Italy | c.229+1G>A(p.G77S) | H | 2 months | M | YES | D,FTT, HSM, AD, AC | A, ET, HBM | BMb | + | ND | death | 6 months | [38] |
| 23 | Italy | c.796G>T (p.G266*) | H | 2 months | F | NO | D, FTT, HSM, AD, AC | A, ET, FB, HBM | BMb | + | ND | death | 5 months | [38] |
| 24 | Croatia | c.419G>A (p.W140*) | H | 4 months | F | YES | D, V, FTT, HSM, AD, AC | ND | ND | + | ND | death | 4 months | [38] |
| 25 | China | c.796G>T (p.G266*) | H | 5 months | F | ND | HSM, AD, AC | A, ET, HTG, CG, HB | Lb，BMb | + | ST | death | 21 Week | [39] |
| 26 | Iranian–Iraqi | c.260G>T (p.G87V) | H | 1 month | M | ND | D, V, FTT, HSM, AC | ND | ND | + | HSCT | death | 6 years | [40] |
| 27 | Englang | c.398delC(p. S133*) | H | 8 days | M | NO | D, V, FTT, HSM | A, ET | ND | + | ERT | Alive at 12 Weeks |  | [18] |
| 28 | Egypt | c.969A＞G (W130*) | H | 1 month | M | YES | FTT, HSM, AD | A, HTG | BMb | ND | ND | death | ND | [41] |
| 29 | Egypt | c.438delC (p.S112*) | H | 3 months | F | YES | FTT, HSM, AD | A, ET, HTG, CG, HBM | BMb | ND | ND | ND | ND | [41] |
| 30 | Egypt | c.969A ＞G(p.W289*) | H | 2.5 months | M | YES | FTT, HSM, AD | A, HTG, HBM | ND | ND | ND | ND | ND | [41] |
| 31 | France | c.193C>T (p.R65*) | H | 1 month | F | NO | D, V, FTT, HSM, AD, AC | A, ET, HTG, HBM | ND | ND | ERT | Alive at 66 months |  | [42] |
| 32 | Mexico | c.894G>C(p. Q298H)/c.1024G>A(p. G342R) | CH | 2.5 months | F | NO | FTT, HSM, AC | A, ET, HTG, CG | BMb | + | ST | death | 5 months | [43] |
| 33 | Iran | c.260G＞T(p.G87V) | H | 2 days | F | ND | HM, AC | ND | ND | ND | BMT | death | 6 months | [44] |
| 34 | Turkish | c:260G>T(p.G87V) | H | 2 months | F | YES | D, V, FTT, HSM, AD | A, ET | BMb | + | ST | death | 3 months | [45] |
| 35 | India | c. 1180_1184del(p. Leu394GluextTer) | H | 75 days | M | NO | V, FTT, HSM, AD, AC | A, HTG | BMb | + | ST | ND | ND | [46] |
| 36 | Spain | c.398delC (p. S133*) | H | 2 months | ND | YES | V, HSM, AC | ND | ND | + | ND | ND | ND | [14] |
| 37 | Spain | c.894G>C (p. Q298H) | H | 45 days | ND | ND | FTT, HSM, AD, AC | ND | ND | + | ND | ND | ND | [14] |
| 38 | Spain | c.894G>C (p. Q298H) | H | 1 month | ND | ND | D, V, FTT, HSM, AD | ND | ND | + | ND | ND | ND | [14] |
| 39 | Spain | c.966+2T>G (IVS9+2T>G) | H | 15 days | ND | YES | D, V, FTT, HSM | ND | ND | + | ND | ND | ND | [14] |
| 40 | Spain | c.966+2T>G (IVS9+2T>G) | H | 1 month | ND | YES | V, FTT, HSM, AC | ND | ND | + | ND | ND | ND | [14] |
| 41 | Spain | c.966+2T>G (IVS9+2T>G) | H | ND | ND | ND | ND | ND | ND | + | ND | ND | ND | [14] |
| 42 | Spain | c.966+2T>G (IVS9+2T>G) | H | 15 days | ND | ND | D, HSM, AD, AC | HTG | ND | + | ND | ND | ND | [14] |
| 43 | Spain | c.966+2T>G (IVS9+2T>G) | H | 2 months | ND | ND | HSM, AC | ND | ND | + | ND | ND | ND | [14] |
| 44 | Spain | c.966+2T>G (IVS9+2T>G) | H | 41 days | ND | YES | D, V, FTT, AC | ND | ND | + | ND | ND | ND | [14] |
| 45 | Spain | c.966+2T>G (IVS9+2T>G) | H | 2 months | ND | YES | D, FTT, HM, AC | ND | ND | + | ND | ND | ND | [14] |
| 46 | Spain | c.966+2T>G (IVS9+2T>G) | H | 15 days | ND | YES | V, FTT, HSM, AD, AC | ND | ND | + | ND | ND | ND | [14] |
| 47 | Spain | c.966+2T>G (IVS9+2T>G) | H | 4 months | ND | ND | ND | ND | ND | + | ND | ND | ND | [14] |
| 48 | Spain | c.966+2T>G (IVS9+2T>G) | H | 25 days | ND | ND | ND | ND | ND | + | ND | ND | ND | [14] |
| 49 | Portugal | c.966+2T>G (IVS9+2T>G) | H | 2 months | M | YES | FTT, HSM, AD, AC | A, ET, HTG, CG, HB, HBM | BMb | + | ERT | death | 5.5 months | [47] |
| 50 | Poland | c.509C > A(p.S103R)/c.796G > T(p.G266X) | CH | 2 months | F | ND | V, FTT, HSM, AD, AC | A, ET, HTG, CG, HB, HBM | BMb | + | ERT | death | 6 months | [47] |
| 51 | Japan | c.169C>G(p. Y22*) | H | 30 days | F | YES | D, V, HSM, AD, AC | A, ET, HTG, HB | Lb，BMb | + | ND | death | 114 days | [48] |
| 52 | Tunisia | c.153C > A (p.Y51*) | H | 3 months | F | YES | FTT, HSM, AD, AC | A, ET, HTG, CG, HB | BMb | ND | ST | death | 4 months | [49] |
| 53 | India | c.676–2A > T /(p.D226Y ) | H | 2 Weeks | F | YES | D, V, FTT, HSM, AD, AC | A, ET, HTG, CG, HB, HBM | BMb | ND | ND | death | 17 weeks | [50] |
| 54 | India | c.193C>T(p.R65*) | H | 1 month | M | ND | FTT, HSM | A, ET, HTG, CG, HB | BMb | ND | ND | death | 2 months | [51] |
| 55 | Ghana | c.358A>C(p.S120R)/c.428+1G>T(p.S143I ) | CH | 6 months | M | NO | D, FTT, HSM, AD, AC | A, ET, HTG | Lb，BMb | ND | ERT | death | 17 months | [52] |
| 56 | Saudi Arabia | chr.10 c.(428 + 1_967-1)_(*1_?)del | H | 4 months | M | YES | FTT, HSM, AD, AC | A, ET, HTG, CG, HB, HBM | BMb | + | ST | death | 4 months | [11] |
| 57 | Englang | chr10:90,947,44991,060,391 (112kb) (hg19) | H | 2 months | ND | ND | D, V, FTT | ET | ND | + | ERT, HSCT | Alive at 63 months |  | [29] |
| 58 | Englang | chr10:90,947,44991,060,391 (112kb) (hg19) | H | 1 Week | ND | ND | ND | ET | ND | + | ERT, HSCT | Alive at 77 months |  | [29] |
| 59 | Englang | c.684delT (p.Phe22Leufs*13) | H | 12 Weeks | ND | ND | HSM | A, ET | ND | + | ERT, HSCT | Alive at 32 months |  | [29] |
| 60 | Englang | chr10:90,947,44991,060,391 (112kb) (hg19) | H | 3 months | ND | ND | D, FTT, HM | ET | ND | + | ERT, HSCT | Alive at 58 months |  | [29] |
| 61 | France | c.538G>A(p.G180S)/c.481delA(p.Asn161Ilefs*19) | CH | 2 months | M | NO | D, V, FTT, HSM, AD, AC | A, ET, HTG | ND | + | ERT | Alive at 122 months |  | [28] |
| 62 | Ivory Coast | c.676-2A>G(p. D226N ) | H | 1 month | M | YES | D, V, FTT, HM, AD, AC | ET | ND | + | ERT | Alive at 84 months |  | [28] |
| 63 | Maroc | c.429-1G>C(p. S143R ) | H | 1 month | F | YES | V, FTT, HM, AD, AC | ND | ND | ND | ERT | Alive at 38 months |  | [28] |
| 64 | Turkish | c.419G>C(p.W140S) | H | 1 month | F | YES | D, HSM, AD, AC | ND | ND | ND | ERT | Alive at 85 months |  | [28] |
| 65 | Eritrea | c.260G>T(p.G87V) | H | 2 months | F | YES | D, V, FTT, HSM, AD, AC | A, ET, HTG, CG, HBM | ND | ND | ERT | Alive at 16 months |  | [28] |
| 66 | America | c. 350_351insCC(p. Met117fs)/c.602G>A(p. G201D) | CH | 2 months | F | ND | V, FTT, HSM, AD, AC | A, ET, HTG | BMb | + | ERT | Alive at 30 months |  | [53] |
| 67 | Egypt | c.260G>T (p.G87V) | H | 2 months | M | YES | D, V, FTT, HSM, AD, AC | A, ET, HTG | ND | ND | ND | death | 4 months | [54] |
| 68 | Egypt | c.260G>T (p.G87V) | H | 2 months | F | YES | D, V, FTT, HSM, AD, AC | A, ET, HTG | ND | ND | ND | death | 3 months | [54] |
| 69 | Egypt | c.1055_1057del(p. Asp352del) | H | 1 month | F | YES | D, V, FTT, HSM, AD, AC | A, ET, HTG, HBM | ND | ND | ND | death | 4 months | [54] |
| 70 | Egypt | c.1055_1057del(p. Asp352del) | H | 1 month | F | YES | D, V, FTT, HSM, AD, AC | A, ET, HTG, HBM | ND | ND | ND | death | 3 months | [54] |
| 71 | Egypt | c.1122 T>G (p. H374Q) | H | 1 month | M | YES | D, V, FTT, HSM, AD, AC | A, ET, HTG, HBM | ND | ND | ND | death | 5 months | [54] |
| 72 | Iran | c. G353A＞G (p.G118D) | H | 4.5 months | M | YES | D, HSM, AD | A, ET, HTG, CG, HB, HBM | BMb | ND | ST | death | 5 months | [6] |
| 73 | Spain | c.966 + 2T > G (IVS9+2T>G) | H | 3.5 months | F | ND | HSM, AD | A, ET, HTG | ND | + | ERT | Alive at 6 months |  | [55] |
| 74 | Spain | c.966 + 2T > G (IVS9+2T>G) | H | 6 Weeks | M | ND | V, FTT, HSM, AC | ET | ND | + | ERT | Alive at 5 months |  | [55] |
| 75 | Spain | chr10:90,947,44991,060,391 (112kb) (hg19) | H | 2.5 months | M | ND | D, V, HSM, AD | A, HTG, HBM | Lb | + | ERT | Alive at 11 months |  | [55] |
| 76 | Englang | c.824C>T (p.S275F) | H | 4 months | F | NO | FTT, HSM, AD | A, ET, HTG, CG | ND | + | ERT, BMT | death | 15 months | [56] |

Abbreviations: H, homozygote; CH, compound heterozygous; M, male; F, famale; D, diarrhea; V, vomitting; FTT, failure to thrive; HM, hepatomegary; SM, splenomegaly; HSM, hepatosplenomegaly; AC, adrenal calcifications; AD, abdominal distension; A, anemia; ET, elevated transaminases; HTG, hypertriglyceridemia; CG, coagulopathy; HB, hyperbilirubin; HBM, Hypoalbuminemia; Lb, liver biopsy, BMb, bone marrow biopsy; ST, supportive therapy; ERT, enzyme replacement therapy; BMT, bone marrow transplant; UMCBT, unrelated mbilical cord blood transplantation; HSCT, hematopoietic stem cell transplantation, RLALa, reduced LAL activity; +, have; ND, no data.
